# Impact of Treadmill Running on Circulating Cortisol Concentrations in Clinically Healthy Dogs

**DOI:** 10.3390/ani15081076

**Published:** 2025-04-08

**Authors:** Jennifer S. Eiermann, Laureen M. Peters, Bérénice Lutz

**Affiliations:** 1 Division of Small Animal Internal Medicine, Department of Veterinary Medicine, Vetsuisse Faculty, University of Bern, 3012 Bern, Switzerland; jennifer.eiermann@unibe.ch; 2 Clinical Diagnostic Laboratory, Department of Clinical Veterinary Medicine, Vetsuisse Faculty, University of Bern, 3012 Bern, Switzerland; laureen.peters@unibe.ch

**Keywords:** adrenocorticotropic hormone stimulation, adrenal testing, exercise, hypoadrenocorticism, stress response

## Abstract

Basal cortisol concentration is used to screen for hypoadrenocorticism (HAD) in dogs with chronic gastrointestinal disease, but low values often lead to unnecessary ACTH stimulation tests. This study evaluated treadmill exercise as a potential alternative method for stimulating cortisol release. Nineteen healthy pet dogs (mean age: 5.2 years, median weight: 23.4 kg) were included. After a baseline blood draw (T0), dogs were acclimated to a treadmill with treats to minimize stress. They then walked and trotted for 10 min, followed by a 30 min rest before a second blood draw (T1). Pre- and post-treadmill exercise cortisol levels were compared using a randomization (permutation) test for paired data. Median pre-exercise serum cortisol was 1.36 μg/dL (IQR: 0.9–2.095), and post-exercise was 1.1 μg/dL (IQR: 0.895–1.585), with no significant difference (*p* = 0.0915). After exercise cortisol increased in 9 of 19 and decreased in 10 of 19 dogs. These findings suggest that treadmill exercise for 10 min does not reliably stimulate cortisol release, assessed 30 min post-exercise. Basal cortisol concentrations in this cohort of clinically healthy dogs were lower than previously described.

## Simple Summary

Dogs with chronic vomiting and diarrhea are frequently screened for hypoadrenocorticism (HAD) by measuring basal cortisol concentrations. Whenever the cortisol concentration is above 2 μg/dL, HAD can be excluded. The goal of this study was to assess whether running on a treadmill could raise such circulating cortisol concentrations in clinically healthy dogs. For this purpose, nineteen healthy pet dogs were included, and after a baseline blood draw (T0), the dogs were acclimated to a treadmill with treats to minimize stress. They then walked and trotted for 10 min, followed by a 30 min rest before a second blood draw (T1). There was no significant difference between pre- and post-exercise cortisol concentrations. Cortisol concentration increased after treadmill running in 9 and decreased in 10 out of 19 dogs. Based on these findings treadmill exercise does not reliably stimulate cortisol release.

## 1. Introduction

Hypoadrenocorticism (HAD) is a relatively uncommon endocrinological disease in dogs, with an estimated prevalence of 0.06 to 0.28% [1]. Certain breeds, such as the Standard Poodle, Nova Scotia Duck Tolling Retriever, and the Portuguese Water Dog, may have a higher prevalence due to heritability [2]. Affected dogs often exhibit episodic gastrointestinal (GI) signs, including diarrhea and vomiting, as well as other non-specific signs like apathy, dehydration, polyuria, polydipsia, weight loss, or shivering [2]. Thus, the exclusion of hypoadrenocorticism as a cause for chronic gastrointestinal signs is of the utmost importance [3].

Numerous studies have evaluated basal cortisol cut-off values to exclude hypoadrenocorticism in dogs with chronic GI signs [4,5,6,7,8]. A basal cortisol concentration above 2 µg/dL has a sensitivity of 100% for ruling out the disease [4,5,6,9], albeit with relatively low specificity (63.3%) [4,5]. As a result, whenever a basal cortisol concentration yields a value at or below 2 µg/dL, an adrenocorticotropic hormone (ACTH) stimulation test (ACTH STIM) is required to confirm or exclude hypoadrenocorticism [3]. This process can lead to additional veterinary visits and increased costs for dog caregivers. Furthermore, over the last years, the cost of synthetic ACTH has dramatically increased in the United States, and there are ongoing issues with availability in Europe [5], which are exacerbated by the COVID-19 pandemic [10,11]. Thus, alternative methods for stimulating and documenting cortisol release in dogs are desirable.

Cortisol secretion follows circadian and ultradian rhythms [12]. Additionally, in the case of stress, either psychological or physiological, the hypothalamic–pituitary–axis is activated and, ultimately, ACTH is released into the blood stream, which stimulates synthetization and the release of cortisol from the adrenal glands [13].

Various studies have explored whether other factors, such as auditory stimuli, acupuncture, or restraint can increase basal cortisol levels in healthy dogs. However, no statistically significant increases in cortisol concentrations were observed in these studies [14,15,16,17,18,19,20,21,22,23]. On the other hand, research on canine transport stress found that blood and salivary cortisol concentrations were significantly higher after ground and air transportation [22], suggesting that stressful events can impact cortisol levels.

In humans, various studies have demonstrated that exercise can elevate circulating cortisol levels, particularly when the exercise intensity is high [24,25]. Despite this, the effect of short-term exercise on cortisol levels in clinically healthy dogs has not been evaluated. This study, therefore, primarily aimed to evaluate the effect of short-term exercise on circulating cortisol concentrations in clinically healthy, privately owned dogs. A secondary aim of the study was to describe the resting cortisol concentration in healthy dogs. We hypothesized that short-term exercise would significantly increase circulating cortisol concentrations in clinically healthy, privately owned dogs.

## 2. Materials and Methods

### 2.1. Study Population

This study protocol was approved by the Institutional Review Board of the Swiss Welfare Committee. Written consent for participation in the study was obtained in all cases.

Dogs were pets, recruited from within the network of the small animal clinic of the University of Bern. Overall, dogs were included if they were between one and ten years of age, as well as >4 kg in weight, if they had no systemic clinical signs in the preceding 4 weeks, an unremarkable history, and a normal clinical examination. Dogs that received any medication, except for routine preventative healthcare, within the preceding 4 weeks, insufficiently trained to undergo blood sampling, or unduly stressed by repeated blood sampling or by running on the treadmill were excluded. Dogs receiving glucocorticoids in the previous 90 days, either orally or topically, were ineligible. Complete blood count (CBC) and biochemistry analyses (e.g., electrolytes, glucose, proteins, kidney values and liver enzymes, bilirubin, cholesterol, triglycerides, 1,2-o-dilauryl-rac-glycreo-3-gludaric acid (DGGR) lipase, and c-reactive protein) were performed, and dogs were excluded from the study if they had clinically relevant changes in either the CBC or biochemistry.

Initially, twenty-one dogs were recruited for this study.

### 2.2. Study Protocol

Dogs were prospectively enrolled from September 2024 to October 2024, and the study was performed at the small animal hospital of the University of Bern.

To avoid waiting times at the hospital and, therefore, to reduce stress, the eligibility criteria were checked and history recorded before undergoing the study protocol. Eligible dogs were seen upon appointment and were immediately brought to the examination area to avoid unnecessary stress. Dogs were accompanied by their caregivers at all times. All samples were collected in the afternoon, as the room and treadmill were utilized for clinical purposes in the morning.

Following initial blood sampling (T0), dogs were introduced to the treadmill so they could explore the installation for several minutes, followed by a complete physical examination by a single experienced, Fear-Free-certified veterinarian (JE). Dogs were always handled according to the principles of the Fear Free Pets Program © (Fear Free, LLC, Nashville, TN, USA).

After this, the dogs were asked to walk on the treadmill (Fit Fur Life ©, Surrey, UK). The velocity of the treadmill was gradually increased to achieve a trotting gait after 2.5 min at the most, and the dogs were on the treadmill for a total duration of 10 min (walk and trot). The velocity of the treadmill was adjusted individually to achieve a trot, based on visual inspection of the dog’s gait by the investigator (JE). If the dogs struggled to keep up with the treadmill or were distracted, in total two short stops of the treadmill were allowed for a maximum duration of 30 s each. After the end of the running period, dogs were allowed to rest for 30 min, as a meta-analysis performed on human medicine has shown that circulating cortisol levels in plasma and saliva peak 21 to 40 min after the onset of psychological stress [26]. At the end of the 30 min waiting period, an additional blood draw was performed (T1). In line with Fear Free Handling protocols, dogs were allowed to eat treats during blood draws and when running on the treadmill. Dogs were not required to be fasted.

### 2.3. Sample Collection and Processing

At the first (T0) and second blood draws (T1), approximately 5 mL and 2 mL of blood, respectively, were collected from a jugular or cephalic vein, using as little restraint as possible. Blood was immediately placed into serum tubes with a clot activator, Li-heparin, and ethylene diamine tetraacetic acid (EDTA) tubes. CBC and biochemistry analyses were performed on sample T0. Serum was allowed to clot at room temperature for approximately 15 min, after which the tubes were centrifuged at 1500× *g* for 10 min (Universal 320, Hettich AG, Bäch, Switzerland) before pipetting the supernatant into plain tubes.

### 2.4. Measurement of Serum Cortisol Concentrations

Serum samples were shipped overnight to the IDEXX Diavet AG Laboratories (Freienbach, Switzerland), after a maximum refrigerated storage time of 72 h. Cortisol concentrations were measured using a competitive chemiluminescence immunoassay (IMMULITE 2000XPi; Siemens Healthcare Diagnostics, Deerfield, IL, USA), previously validated for canine serum. The lower limit of detection for the cortisol concentrations was <0.50 µg/dL; whenever the value was below this limit, to allow for statistical analysis, the value was recorded as 0.49 µg/dL.

### 2.5. Statistical Analysis

The statistical analysis was completed using commercially available software (NCSS 2024 Statistical Software. NCSS, LLC. Kaysville, UT, USA, ncss.com/software/ncss).

A sample size calculation was performed based on retrospective data collected at the Small Animal Clinic of the University of Bern. Briefly, based on ACTH STIM in dogs with a clinicopathological suspicion of HAC leading to a mean increase in cortisol concentrations of 10 µg/dL (SD: 8.05; unpublished data), we hypothesized that exercise would lead to a sub-maximal cortisol stimulation of approximately 25% of the stimulation capacity of ACTH-STIM, i.e., 2.5 µg/dL. With alpha set at 0.05 and power at 0.8, we calculated a sample size of 38 dogs for comparison of paired differences. Because of the lack of a significant difference after inclusion of half the calculated sample size, it was decided to discontinue enrolment, in adherence to the 3R principles in animal research [27].

Descriptive statistics were used for population characteristics. Data distributions were checked for normality by examining histograms and by the Shapiro–Wilk test. Non-normally distributed values are presented as the median (interquartile range (IQR)), and normally distributed values are presented as the mean (standard deviation (SD)). To test for differences between the median pre- and post-running cortisol concentrations, a randomization (permutation) test for paired data was used. Statistical significance was set at *p* < 0.05.

## 3. Results

### 3.1. Study Population

Initially, twenty-one dogs were recruited for this study. One dog did not finish the complete study protocol, as he was unduly stressed by running on the treadmill, and one dog was excluded after completing the study protocol because of azotemia and low-urine-specific gravity after performing the study protocol, leaving a total of 19 dogs included in the study. An overview of the population is provided in Table 1.

Dogs were between one and ten years of age (mean: 5.2 years, SD: 3.2). Ten dogs were female, nine dogs were male. Of the female dogs, eight were neutered and two were intact. Among the male dogs, three were neutered and six were intact. Five dogs were mixed-breed dogs and 14 were purebred, belonging to nine different breeds with the most common breeds being German Shepherd (n = 4) and Dachshund (n = 2). The median body condition score was 5 out of 9 (IQR: 5–5.5). The median weight was 23.4 kg (IQR: 13.5–30.6).

### 3.2. Acceptance and Velocity of the Treadmill

Thirteen dogs were able to run on the treadmill for 10 min without any pause. Two dogs had a single pause, and four dogs had two pauses during the 10 min interval. All dogs that had at least one pause continued to walk in a relaxed manner after the re-initiation of the treadmill after the pause. Sixteen dogs walked freely on the treadmill and showed no signs of stress during the exercise, and three dogs were subjectively mildly stressed by the treadmill but still managed to finish the study protocol without being unduly stressed.

The median velocity of the treadmill needed to achieve a trotting gait was 5.6 m/s (IQR: 4.8–6.35, range: 2.7–7.4).

### 3.3. Paired Serum Cortisol Concentrations

**Figure 1:**
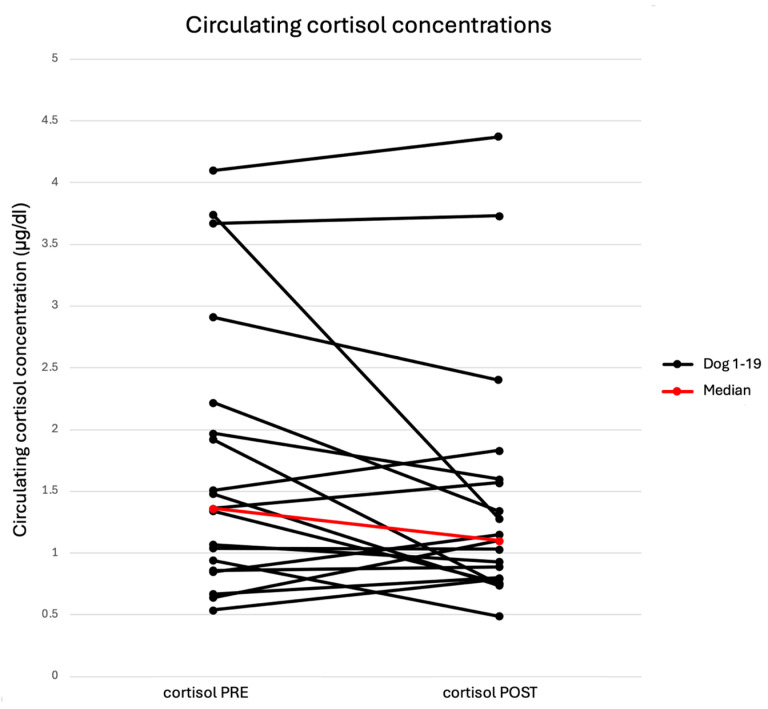
Relationship between individual paired pre- and post-running cortisol concentrations, as well as the median.

The median resting serum cortisol concentration was 1.36 µg/dL (IQR: 0.9–2.095, range: 0.54–4.1). The median post-running serum concentration was 1.1 µg/dL (IQR: 0.895−1.585, range: 0.49–4.37). There was no significant difference between median pre- and post-running serum cortisol concentrations (*p* = 0.0915). Cortisol concentrations increased in 9/19 and decreased in 10/19 paired values after treadmill running. The relationship between individual pre- and post-running serum cortisol concentrations is depicted in Figure 1.

## 4. Discussion

This study is, to our knowledge, the first to evaluate the impact of running on a treadmill on circulating cortisol concentrations in clinically healthy dogs, and it did not reveal a significant difference between pre- and post-running concentrations.

The aim of this study was to determine whether short-duration exercise could elevate cortisol concentrations, which are commonly measured to screen for HAD in dogs. The established cut-off of 2 μg/dL offers a high sensitivity but only moderate specificity, which leads to numerous dogs with non-adrenal illnesses being subjected to additional ACTH-STIM testing.

In Alaskan sled dogs, urinary cortisol concentrations were significant higher in dogs that ran long distances (between 160 and 560 km) compared to dogs that did not perform any exercise [18]. The overall duration, distance, and intensity of the exercise by these dogs was much greater than in the present study, which raises the question of whether the exercise performed on the treadmill was not intense enough for a significant increase in the serum cortisol concentration to be noted. Another factor to be considered is that although urinary cortisol concentrations have been shown to generally correlate well with plasma cortisol concentrations [28], they may not respond to physical activity in the same manner. An older study showed that plasma levels of ACTH and cortisol increased with the duration of exercise and, additionally, increased faster with a higher level of exercise intensity [29]. This could also indicate that either the intensity of the exercise in this study was not high enough or the duration of the exercise was too short.

Post-cortisol concentration was assessed after a 30 min rest period, as a meta-analysis performed in human medicine has shown that circulating cortisol levels in both plasma and saliva peak 21 to 40 min after the onset of psychological stress [26]. Based on these results, we extrapolated that plasma cortisol levels in dogs would also peak at approximately same time point after exercise. In a short communication published on serum cortisol changes in dogs responding to thunder, cortisol levels were found to peak approximately 15 min after exposure, returning to baseline within 30 to 60 min [19]. Future studies should consider sampling dogs at 15, 30, 45, and 60 min post-exercise to ensure detection of peak serum cortisol levels.

The stress induced by treadmill running differs from psychological stress, such as that caused by loud noises or thunder. This distinction may contribute to variations in the kinetics of cortisol release depending on the type of stress. It, therefore, remains possible that serum cortisol values did increase in the dogs running on the treadmill, but that because of a suboptimal timing of the sampling, the increase was missed in this study.

Stressful events such as air transport and exposure to loud noises have shown to lead to increased salivary cortisol concentrations in dogs [14,19,30]. In a study evaluating exposure to multiple stressors, upon exposure of dogs to a falling bag or to electric shocks, the dogs showed a similar increase in salivary cortisol concentrations than when exposed to loud noise, but a pronounced variation among individuals was found, which lead to nonsignificant results [14]. In the present study, cortisol increased and decreased in a similar proportion of dogs post-exercise, supporting this pronounced inter-individual variation. As with urinary cortisol, salivary cortisol concentrations have been shown to correlate well with serum cortisol concentrations [31].

In between the first blood draw and running on the treadmill, the dogs underwent a clinical exam, which could have led to some stress in the dogs, falsely increasing cortisol concentrations. Because there was no significant difference between the pre- and post-exercise cortisol concentration, this, however, seems rather unlikely. All dogs were examined by the same veterinarian, who is certified in Fear Free Handling, and care was taken to avoid any unnecessary stress.

An *a priori* power study suggests that at least 38 dogs need to be included to allow for a sufficient power of the study. In accordance with 3R principles [27], after half of the study population was included, preliminary data were evaluated, and because of the obvious lack of difference between pre- and post-cortisol concentrations, it was decided to stop data collection. This might have led to a type II error; however, based on the data from the first 19 dogs, inclusion of the initially calculated 38 individuals would have been unlikely to result in a statistically significant difference. Thus, it was considered unethical to test additional dogs, and recruitment was discontinued.

A secondary aim of our study was to report basal cortisol concentrations in clinically healthy dogs. The median of our study population was below the threshold of 2 μg/dL, which is currently used as a cut-off value to exclude HAD in dogs with chronic gastrointestinal disease. Conversely, all but two dogs had both cortisol concentrations within the reference interval provided by the laboratory that performed the measurements. In the two dogs, one of both (i.e., once T0 and once T1) measurements was within the reference interval. Although the population included in our study does not necessarily represent the population of dogs that would be screened for HAD, only 5 out of 18 dogs in our study had cortisol concentrations above 2 μg/dL, and the remaining 13 dogs might have been, in a clinical setting, subjected to an ACTH STIM. This once again highlights the need for improved exclusion testing for HAD.

All dogs involved in this study were recruited through a personal network and most caregivers were associated in some way with the small animal hospital, so the majority of the dogs were well acclimated to this environment. This may have led to very low resting cortisol concentrations. An earlier study performed evaluating serum cortisol concentrations in dogs undergoing auditory stimuli, however, found median resting cortisol values of 2.3 μg/dL or higher depending on the study group [20]. Those dogs were also acclimated to the environment of the animal hospital. Why the cortisol concentration in the present study was lower than previously described is not fully clear. In humans, cortisol secretion follows circadian and ultradian rhythms, with peak secretion levels in the mornings [12,13]. Studies in dogs on the circadian and ultradian rhythms of cortisol secretion showed conflicting results, with some studies showing the highest cortisol concentrations between 10 a.m. and 2 p.m. [32,33], and some studies showing similar concentrations during the whole day [34]. In the present study, all samples were collected after 12 p.m., which might, at least in part, explain the lower circulating cortisol concentrations compared to the previous study, where samples were also collected in the morning [20], if cortisol concentrations were already lowering in the afternoon.

This study had several limitations. Cortisol concentrations were only measured once 30 min after the end of the exercise and not in five-minute intervals after the exercise in order to minimize stress for the patient and the amount of blood collected. Because of this, an earlier peak in serum cortisol concentrations could have been missed. The velocity of the treadmill was individually adjusted to allow the dogs to achieve a trot and no fixed velocity based on weight was used, which might have led to some differences in the actual velocity for each individual dog.

Future research focusing on other types of stressors that might lead to an increase in cortisol levels could be of interest to identify alternatives to the ACTH STIM for the exclusion of HAD in dogs with chronic gastrointestinal symptoms and a low level of suspicion.

## 5. Conclusions

Running on a treadmill for 10 min does not have a significant effect on the circulating cortisol concentration measured 30 min post-exercise in clinically healthy dogs. Thus, it does not appear promising as a suitable alternative to ACTH STIM to exclude HAD. Basal cortisol concentrations in this cohort of clinically healthy dogs were lower than previously described.

## Figures and Tables

**Table 1:** Overview of the study population.

Dog No.	Breed	Age (years)	Sex	Weight (kg)
1	Mix Breed	6	Female neutered	18
2	Siberian Husky	10	Male entire	29
3	Border Collie	6	Female neutered	18
4	Miniature American Shepherd	4	Female neutered	10
5	Mix Breed	6	Female neutered	32
6	Mix Breed	10	Male neutered	24
7	Dachshund	3	Female neutered	4
8	Labrador Retriever	10	Female neutered	31
9	Mix Breed	1	Female neutered	14
10	American Staffordshire Terrier	2	Male entire	25
11	German Shepherd	6	Female entire	34
12	German Shepherd	8	Male entire	33
13	Dachshund	2	Male entire	10
14	French Bulldog	1	Female neutered	13
15	German Shepherd	3	Female entire	20
16	German Shepherd	4	Female entire	39
17	Jack Russel Terrier	6	Male entire	11
18	Mix Breed	1	Male entire	23
19	Mix Breed	10	Male neutered	22

## Data Availability

The data that support the findings of this study are openly available from figshare at https://figshare.com/s/2c0da3a1253d5563bba8.

## Abbreviations

The following abbreviations are used in this manuscript:

ACTH	Adrenocorticotropic hormone
ACTH STIM	Adrenocorticotropic hormone stimulation test
CBC	Complete blood count
EDTA	Ethylene diamine tetraacetic acid
IQR	Interquartile range
HAD	Hypoadrenocorticism
SD	Standard deviation

## References

[1] Kelch W.J., Lynn R.C., Smith C.A., New J.C. (1998). Canine hypoadrenocorticism (Addison’s disease). Compend. Contin. Educ. Pract. Vet..

[2] Scott-Moncrieff J.C., Feldman E.C., Nelson R.W., Reusch C.E., Scott-Moncrieff J.C.R. (2015). Hypoadrenocorticism. Canine and Feline Endocrinology.

[3] James Hall E., Day M.J., Ettinger S.J., Feldman E.C., Côté E. (2017). Diseases of the Small Intestine. Textbook of Veterinary Internal Medicine Disease of the Dog and Cat.

[4] Bovens C., Tennant K., Reeve J., Murphy K.F. (2014). Basal Serum Cortisol Concentration as a Screening Test for Hypoadrenocorticism in Dogs. J. Vet. Intern. Med..

[5] Lennon E.M., Boyle T.E., Hutchins R.G., Friedenthal A., Correa M.T., Bissett S.A., Moses L.S., Papich M.G., Birkenheuer A.J. (2007). Use of basal serum or plasma cortisol concentrations to rule out a diagnosis of hypoadrenocorticism in dogs: 123 cases (2000–2005). J. Am. Vet. Med. Assoc..

[6] Feldman E.C., Peterson M.E. (1984). Hypoadrenocorticism. Vet. Clin. N. Am. Small Anim. Pract..

[7] Van Lanen K., Sande A. (2014). Canine Hypoadrenocorticism: Pathogenesis, Diagnosis, and Treatment. Top. Companion Anim. Med..

[8] Lathan P., Thompson A. (2018). Management of hypoadrenocorticism (Addison’s disease) in dogs. Vet. Med. Res. Rep..

[9] Botsford A., Behrend E.N., Kemppainen R.J., Gaillard P.R., Oprandy F., Lee H.P. (2018). Low-dose ACTH stimulation testing in dogs suspected of hypoadrenocorticism. J. Vet. Intern. Med..

[10] Duffin J. (2020). An activist history of drug shortages and its silos. Lancet.

[11] Ramakrishnan M., Poojari P.G., Rashid M., Nair S., Pulikkel Chandran V., Thunga G. (2023). Impact of COVID-19 pandemic on medicine supply chain for patients with chronic diseases: Experiences of the community pharmacists. Clin. Epidemiol. Glob. Health.

[12] Timmermans S., Souffriau J., Libert C. (2019). A general introduction to glucocorticoid biology. Front. Immunol..

[13] Spiga F., Walker J.J., Terry J.R., Lightman S.L. (2014). HPA axis-rhythms. Compr. Physiol..

[14] Beerda B., Schilder M.B.H., van Hooff J.A.R.A.M., de Vries H.W., Mol J.A. (1998). Behavioural, saliva cortisol and heart rate responses to different types of stimuli in dogs. Appl. Anim. Behav. Sci..

[15] Engeland W.C., Miller P., Gann D.S. (1990). Pituitary-adrenal and adrenomedullary responses to noise in awake dogs. Am. J. Physiol.-Regul. Integr. Comp. Physiol..

[16] Siniscalchi M., Quaranta A., Rogers L.J. (2008). Hemispheric Specialization in Dogs for Processing Different Acoustic Stimuli. PLoS ONE.

[17] Dreschel N.A., Granger D.A. (2005). Physiological and behavioral reactivity to stress in thunderstorm-phobic dogs and their caregivers. Appl. Anim. Behav. Sci..

[18] Durocher L.L., Hinchcliff K.W., Williamson K.K., McKenzie E.C., Holbrook T.C., Willard M., Royer C.M., Davis M.S. (2007). Effect of strenuous exercise on urine concentrations of homovanillic acid, cortisol, and vanillylmandelic acid in sled dogs. Am. J. Vet. Res..

[19] de Souza C.C., Maccariello C.E., Dias D.P., dos Santos Almeida N.A., de Medeiros M.A. (2017). Autonomic, endocrine and behavioural responses to thunder in laboratory and companion dogs. Physiol. Behav..

[20] Gin T.E., Puchot M.L., Cook A.K. (2018). Impact of an auditory stimulus on baseline cortisol concentrations in clinically normal dogs. Domest. Anim. Endocrinol..

[21] Maccariello C.E., de Souza C.C., Morena L., Dias D.P., de Medeiros M.A. (2018). Effects of acupuncture on the heart rate variability, cortisol levels and behavioural response induced by thunder sound in beagles. Physiol. Behav..

[22] Fergestad M.E., Jahr T.H., Krontveit R.I., Skancke E. (2015). Serum concentration of gastrin, cortisol and C-reactive protein in a group of Norwegian sled dogs during training and after endurance racing: A prospective cohort study. Acta Vet Scand..

[23] Angle C.T., Wakshlag J.J., Gillette R.L., Stokol T., Geske S., Adkins T.O., Gregor C. (2009). Hematologic, serum biochemical, and cortisol changes associated with anticipation of exercise and short duration high-intensity exercise in sled dogs. Vet Clin. Pathol..

[24] Hill E.E., Zack E., Battaglini C., Viru M., Viru A., Hackney A.C. (2008). Exercise and circulating Cortisol levels: The intensity threshold effect. J. Endocrinol. Investig..

[25] Athanasiou N., Bogdanis G.C., Mastorakos G. (2023). Endocrine responses of the stress system to different types of exercise. Rev. Endocr. Metab Disord..

[26] Dickerson S.S., Kemeny M.E. (2004). Acute Stressors and Cortisol Responses: A Theoretical Integration and Synthesis of Laboratory Research. Psychol. Bull..

[27] (1960). The Principles of Humane Experimental Technique. Med. J. Aust..

[28] Jones C.A., Refsal K.R., Lippert A.C., Nachreiner R.F., Schwacha M.M. (1990). Changes in adrenal cortisol secretion as reflected in the urinary cortisol/creatinine ratio in dogs. Domest. Anim. Endocrinol..

[29] Radosevich P.M., Nash J.A., Brooks Lacy D., O’Donovan C., Williams P.E., Abumrad N.N. (1989). Effects of low- and high-intensity exercise on plasma and cerebrospinal fluid levels of ir-β-endorphin, ACTH, cortisol, norepinephrine and glucose in the conscious dog. Brain Res..

[30] Bergeron R., Scott S.L., Émond J.-P., Mercier F., Cook N.J., Schaefer A.L. (2002). Physiology and behavior of dogs during air transport. Can. J. Vet. Res..

[31] Wenger-Riggenbach B., Boretti F.S., Quante S., Schellenberg S., Reusch C.E., Sieber-Ruckstuhl N.S. (2010). Salivary Cortisol Concentrations in Healthy Dogs and Dogs with Hypercortisolism. J. Vet. Intern. Med..

[32] Castillo V.A., Cabrera Blatter M.F., Gómez N.V., Sinatra V., Gallelli M.F., Ghersevich M.C. (2009). Diurnal ACTH and plasma cortisol variations in healthy dogs and in those with pituitary-dependent Cushing’s syndrome before and after treatment with retinoic acid. Res. Vet. Sci..

[33] Giannetto C., Fazio F., Assenza A., Alberghina D., Panzera M., Piccione G. (2014). Parallelism of circadian rhythmicity of salivary and serum cortisol concentration in normal dogs. J. Appl. Biomed..

[34] Kolevská J., Brunclík V., Svoboda M. (2003). Circadian Rhythm of Cortisol Secretion in Dogs of Different Daily Activities. Acta Vet. Brno.

